# Urbanization Drives Patterns of Individual Specialization but Not Dietary Diversity Among Coyotes

**DOI:** 10.1002/ece3.74170

**Published:** 2026-08-09

**Authors:** Samantha Erin Sophia Kreling, Ellen M. Reese, Olivia M. Cavalluzzi, Alexandra Renouard, Yasmine Hentati, Robert A. Long, Laura R. Prugh

**Affiliations:** ^1^ School of Environmental and Forest Sciences University of Washington Seattle Washington USA; ^2^ Woodland Park Zoo Seattle Washington USA

**Keywords:** *Canis latrans*, diet, eDNA, individual specialization, metabarcoding

## Abstract

Niche partitioning and resource specialization can lead to spatial heterogeneity in trophic dynamics, but little is known about how these dynamics may change across landscapes of varying complexity. Urban ecosystems are characterized by a wider array of both natural and anthropogenic food sources, which can increase predator densities compared to exurban/rural and wildland areas. Urbanization could thus intensify intraspecific competition and facilitate individual specialization, or relax intraspecific competition if resources are not limited, leading to more generalized diets. To investigate how urbanization affects dietary niche breadth and individual specialization, we used fecal DNA metabarcoding to analyze vertebrate and plant dietary components of 50 coyotes ( *Canis latrans* ) across three landscapes in Washington, USA: the Seattle metropolitan area, Bainbridge Island, and two wildland study sites. Population‐level dietary richness was similar between urban and island coyotes ($\bar{x}$ = 51 and 49 taxa, respectively), but considerably lower for wildland coyotes ($\bar{x}$ = 33 taxa). Across sites, coyotes ate a variety of Rosaceae plants, voles, and rabbits. Deer were an important food source for wildland coyotes, but not for urban or island coyotes. Despite variation in population‐level richness, individual dietary richness was surprisingly consistent within individuals across regions. Urban and exurban island coyotes had the lowest degrees of individual specialization, whereas wildland coyotes exhibited the highest degree of individual specialization. Specialization and dietary diversity may be constrained by home range size and the diversity of prey types available to an individual coyote, highlighting the importance of landscape and prey heterogeneity across landscapes. These findings suggest that urbanization can relax intraspecific competition among predators, whereas individual dietary specialization may have a greater influence on predator–prey dynamics and human‐wildlife conflict in more natural ecosystems with less anthropogenic influence.

## Graphical abstract

Niche partitioning and resource specialization can lead to spatial heterogeneity in trophic dynamics, but little is known about how these dynamics may change across landscapes of varying complexity. Urban ecosystems are characterized by a wider array of both natural and anthropogenic food sources, which can increase predator densities compared to exurban and wildland areas. We found that population level dietary richness was similar between urban and island coyotes ($\bar{x}$ = 51 and 49 taxa, respectively), but considerably lower for wildland coyotes ($\bar{x}$ = 33 taxa), and despite variation in population‐level richness, the diversity of individual diets was surprisingly consistent across regions. Urban coyotes had limited individual specialization, whereas wildland coyotes exhibited the highest individual specialization.

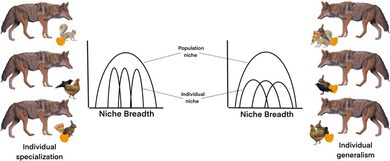

## 1 Introduction

Niche partitioning and resource specialization are key mechanisms by which species reduce inter and intraspecific competition (Alley 1982; Bolnick et al. 2003). The ways in which resources are partitioned among competing individuals can define the success of the population, especially when population density is high or resources are limited (Bolnick et al. 2003). For example, individuals that specialize in singular or limited food types often experience higher foraging success, leading to reduced energy expenditure and higher reproductive success (Bolnick et al. 2003; van den Bosch et al. 2019). While much theoretical work on niche partitioning has been conducted, empirical data is still lacking, especially in novel ecosystems like urban areas (Bolnick et al. 2003). Urban regions can support high densities of human‐ and disturbance‐tolerant wildlife species, such as mesocarnivores (Fischer et al. 2012), because cities are generally characterized by diverse, abundant food driven by anthropogenic influences (Smith et al. 2018; Gámez et al. 2022). Increased food source diversity should provide increased potential for resource partitioning among individuals. This may allow them to specialize on different resources and higher mesocarnivore density in urban areas may increase intra and interspecific competition, facilitating strong resource partitioning. Individuals with high diet diversity can also have differing levels of generalism based on the proportions each item is. However, the degree to which anthropogenic influences alter resource specialization is not well understood and may be highly context dependent (Newsome et al. 2015; Sévêque et al. 2020; Manlick and Newsome 2021). Understanding how these coexistence mechanisms are affected by urbanization is increasingly important as urbanization accelerates worldwide (Sévêque et al. 2020).

**FIGURE 1:**
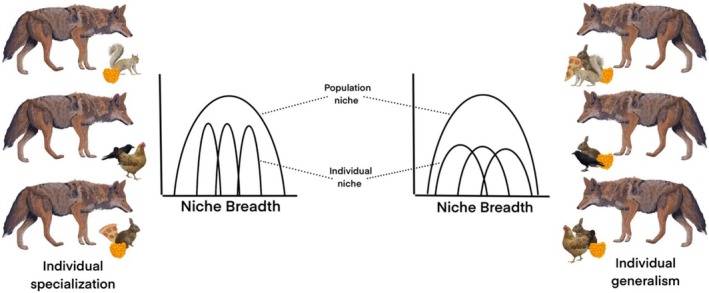
Conceptual figure depicting population‐level dietary generalism coming from high inter‐individual variation and individual specialization (left) versus individual generalism with low inter‐individual variation (right). Conceptual dietary items are displayed in front of each individual coyote.

Coyotes ( *Canis latrans* ) are an important apex predator in urban regions and have had great success during the Anthropocene, doubling their historic range (Hody and Kays 2018). Understanding coyote resource partitioning may thus help to understand their success in these times of immense landscape‐level change, allowing us to apply this knowledge to other species to facilitate adaptation and co‐existence. While coyotes are classic dietary generalists at the population level, consuming a wide variety of plants and animals across a range of urbanization levels (Murray et al. 2015; Poessel, Mock, and Breck 2017; Sugden et al. 2021; Jensen et al. 2022), it is unclear if this population‐level generalism arises because all individuals are generalists or because individuals specialize on different dietary items within the population (Figure 1). In urban areas mesocarnivores populations are denser due to increased food availability (Fischer et al. 2012; Šálek et al. 2014) and opposing strategies for coexistence could emerge (Sévêque et al. 2020). While population dietary niche breadth is expected to be wider in urban areas due to increased resource variety and increased population density, individuals may specialize on certain resources to decrease intraspecific competition (Murray et al. 2015; Sugden et al. 2021). For example, coyotes in Chicago, Illinois had greater dietary variation among individuals than coyotes in surrounding exurban regions (Newsome et al. 2015), while Caspi, Sit, et al. (2025) found increased dietary specialization among urban coyotes compared to rural or wildland coyotes. Alternatively, if resources are plentiful enough that coyote populations are not limited by resource competition, coyotes may consume the most abundant items within their home ranges and have low degrees of individual specialization. Indeed, Larson et al. (2020) found reduced individual variation in urban coyotes and wide niche breadth in suburban areas of Los Angeles, California compared to nearby wildland areas.

However, until recently, inferences regarding effects of urbanization on niche partitioning and individual specialization have been limited by traditional methods of scat analysis that identify diet items based on identification of undigested remains. Advances in genomic methods such as fecal DNA metabarcoding allow for high‐resolution dietary construction (Kartzinel et al. 2015) that is especially advantageous in urban areas where animals may consume foods that are difficult or impossible to identify using traditional methods (De Barba et al. 2013; Forin‐Wiart et al. 2018). Recent studies have highlighted the utility of DNA metabarcoding for determining diets and resource partitioning among carnivore species across varying urbanization levels (Smith et al. 2018; Monterroso et al. 2018; Kluever et al. 2022; Caspi, Serrano, et al. 2025; Caspi, Sit, et al. 2025).

Our objective was to determine how urbanization affects coyote niche breadth and individual diet specialization. We used fecal DNA metabarcoding to generate a comprehensive diet dataset among three distinctive landscape contexts in Washington, USA. Our samples come from the Seattle metropolitan area, an offshore exurban island, and two wildland (low levels of human influence) study sites in northern Washington. If urbanization intensifies intraspecific competition, we expect urban coyotes to have reduced niche breadths and increased individual specialization compared with those in wildland regions. If urbanization relaxes competition, we expect urban coyotes to have wider niche breadths and less individual specialization than their wildland counterparts.

## 2 Methods

### 2.1 Study Region

**FIGURE 2:**
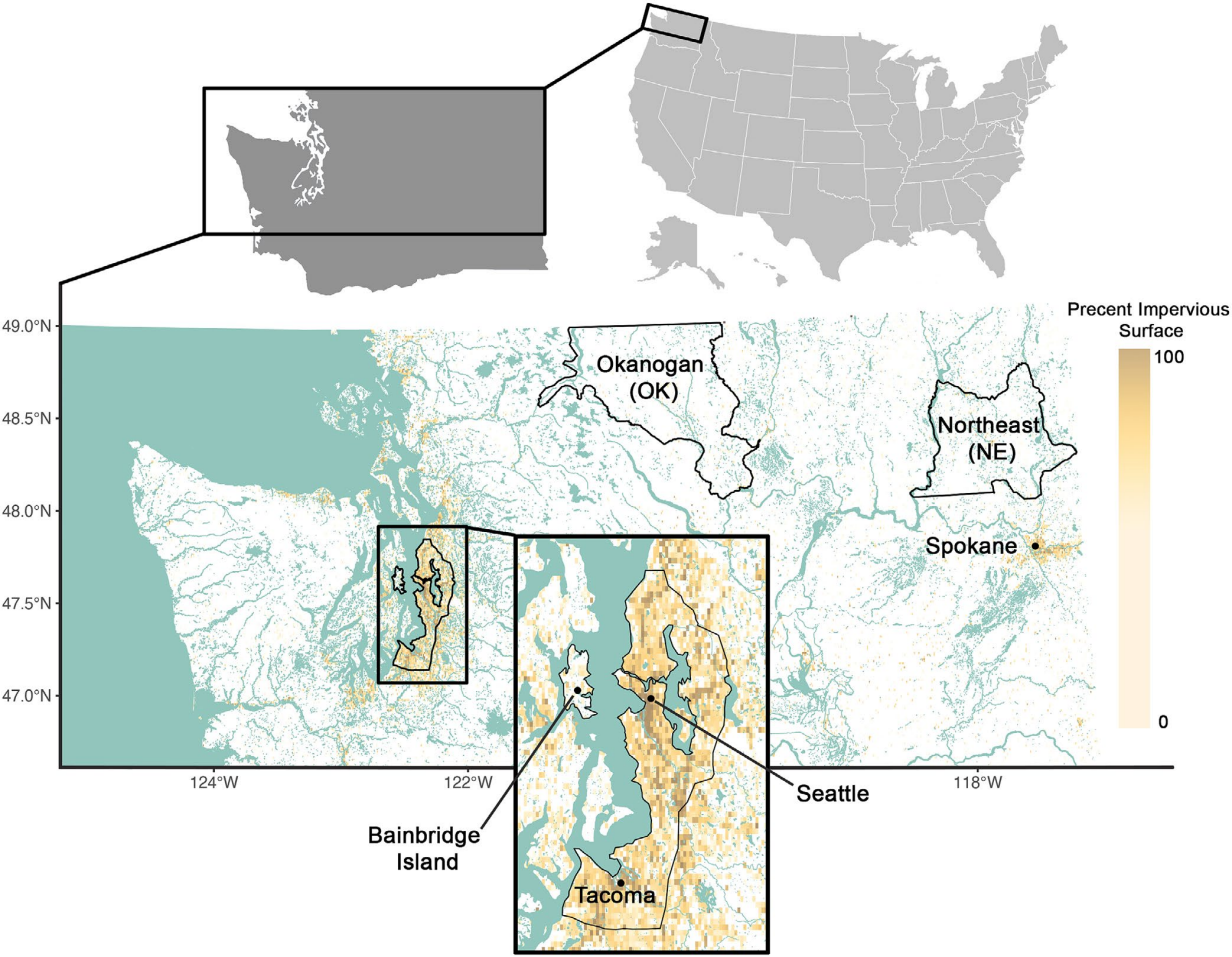
On the top, a map of the Northern portion of the state of Washington state with all study areas outlined in black and a depiction of where the inset is within Washington state, and the United States. Waterways are highlighted in blue. The gradient from yellow to brown represents the percent of impervious surface (NLCD 2019) across the region depicted. In the middle, an inset of the Bainbridge Island and Seattle metropolitan study areas. Major cities are indicated on both maps.

Non‐invasive scat collection was conducted in three study regions ranging from highly urbanized to exurban/rural to natural forested lands within the state of Washington. Urban samples were collected from the Seattle metropolitan area (~1200 km^2^; Figure 2), which is home to over 4 million people and has a human density of 264.5 people/km^2^ (US Census Bureau 2026). The majority of samples come from the urban core of this area such as the cities of Seattle, Tacoma, and Bellevue with population densities of 3592, 3865, and 1752 people/km^2^ respectively (US Census Bureau 2026). This urban region contains a mix of highly developed areas (e.g., downtown Seattle/Bellevue), industrial areas, residential housing, agricultural lands, and natural forest fragment. Roughly 75% of the urbanized areas of Seattle metropolitan area where samples were collected is developed land, 14% is forested, 8% is open water or wetlands, and 2% is agriculture (remainder is open grasslands or barren land; Dewitz 2021). Our island data comes from Bainbridge Island, a rural, exurban island just off the coast of Seattle within the Puget Sound (Figure 2). Bainbridge Island has an area of 72 km^2^ and human density of ~354 people/km^2^. Roughly 11% of this island is developed land and 8% is agricultural, with high levels of tree cover (73%; Bainbridge Island Metro Park and Recreation District 2026). Our wildland samples were collected as part of the Washington Predator Prey Project (https://predatorpreyproject.weebly.com) in Okanagan (OK) county and northeastern Washington (NE). These wildland areas (~3000 km^2^ each) are characterized by low human density (4.5 people/km^2^; Malesis et al. 2024) and > 95% natural land cover, with 3.5% agricultural cover and 1.1% developed areas (Ganz et al. 2024). We analyzed the two wildland areas together because they shared similar landscape characteristics. The Seattle metropolitan area and Bainbridge Island both experience maritime climates with cool, rainy winters and warm, dry summers, whereas the wildland areas have a mountainous continental climate with snowy winters and warm, dry summers.

### 2.2 Scat Collection

Scats were collected from trails within greenspaces across the Seattle metropolitan area 2021–2023, with emphasis on months May–October when precipitation was lower and samples were more likely to yield higher quality DNA. In 2021, green spaces within the city of Seattle were repeatedly sampled on an approximately 2 week basis as reported in Kreling et al. (2023). After 2021, scats were still predominantly collected during the summer, but only a subset of more coyote‐frequented sites was surveyed at a similar 2‐week interval within Seattle and several green spaces throughout the Greater Seattle Region. Sites outside of city limits were sampled on a monthly basis. Scats were collected on our island site in the summer of 2021 on an irregular schedule with some sites only sampled once and others sampled multiple times based on volunteer availability. Scats in the Okanagan and Northeast study regions were systematically collected along dedicated scat transects walked or driven once per month during summer 2018–2020 and winter 2020–2021 as part of the Washington Predator Prey Project. Scats were also collected opportunistically during other field activities during these periods. While there are differences in the time periods that scats were collected at our different study sites, there is overlap among all time periods. Our urban and rural island locations also experience significantly different weather and climatic patterns than our wildland areas, making it unlikely that time‐specific differences in resource availability would be consistent across the areas and limiting the effect that time should have on the results. In all areas, scats were collected with Ziploc bags and then placed within Whirl‐Pak bags, held in coolers or freezers before being transferred to a lab, and frozen at −80°C after removal from the environment until they were thawed to be swabbed for DNA extraction.

### 2.3 DNA Extraction & Microsatellite Genotyping

All laboratory steps except for DNA sequencing and fragment analysis were conducted in the School of Environmental and Forest Sciences (SEFS) Genetics Lab at the University of Washington. To extract DNA, scat samples were thawed and divided into four sections. We swabbed the outer surface of each scat, focusing on the ends where epithelial cells are most likely to be present, to collect DNA for individual coyote genotyping. Using the same swab, we then sampled each internal section to gather DNA from ingested items. Each scat was swabbed with a flocked swab moistened with phosphate‐buffered saline solution. DNA extraction followed the QIAmp DNA Investigator Kit protocol (Qiagen, Hilden, Germany) with minor volume adjustments.

Scats collected from wildland study areas for the Washington Predator Prey Project were screened for predator ID using a mitochondrial species ID panel, allowing coyote scats to be distinguished from other sympatric carnivore species such as wolves ( *Canis lupus* , see Ganz et al. 2023 for details). This panel was initially applied to scats collected from urban areas, but we determined that the collection rate of non‐coyote scats from urban areas was much lower than in the wildland study areas and it was more cost‐effective to screen out non‐coyote scats at the later microsatellite genotyping and DNA metabarcoding steps (see Kreling et al. 2023 for details).

For individual coyote identification, we amplified DNA from the samples at 10 microsatellite loci and two sex ID loci using two multiplexed PCR panels. To minimize genotyping errors, PCR was replicated three times for each sample, following Prugh et al. (2005). The first panel amplified nuclear microsatellite loci FH2001, FH2010, FH2054, FH2088, and FH2328. The second panel amplified CXX2235, FH2096, FH2137, FH2140, FH2159, and two additional loci on the X and Y chromosomes for sex determination (DBX6, DBY7; Prugh et al. 2005; Seddon 2005). Both panels were processed using touchdown‐PCR: an initial 15‐min denaturation at 95°C, followed by 10 touchdown cycles at 94°C for 30 s, 68°C decreasing by 1°C per cycle for 30 s, and 72°C for 45 s. This was followed by 30 cycles at 94°C for 30 s, 58°C for 30 s, and 72°C for 45 s, with a final extension at 60°C for 15 min. After amplification, the PCR plates were sent to Yale's Keck DNA Sequencing Core for fragment analysis using an Applied Biosystems 3130 Series Genetic Analyzer via capillary electrophoresis. Allele sizes were quantified using GeneMapper version 6 (Curie‐Fraser and Shah 2010), and consensus genotypes were created from the replicates following Prugh et al. (2005). Genotypes were compared with those from domestic dog reference samples in Structure (Pritchard et al. 2000) to filter out dog scats or samples contaminated by dogs (e.g., urination in urban areas).

### 2.4 Library Preparation & Sequencing

To analyze coyote diet, we performed PCR amplifications using a multiplex of two primer pairs: 12SV5 for vertebrates (Riaz et al. 2011) and trnL g/h for plants (Taberlet et al. 2007). PCR reactions were 20 μL in volume and contained 0.2 μM each of primer, 10 μL of Qiagen Multiplex PCR Master Mix, 4 μL of Q solution, and 2 μL of extracted DNA. The thermal cycling protocol consisted of an initial denaturation step of 95°C for 15 min, followed by 40 cycles of 94°C for 30 s, 54°C for 90 s, and 72°C for 60 s, followed by a final elongation step of 72°C for 15 min. Each sample was PCR amplified in triplicate and each PCR plate contained control samples to monitor for contamination. PCR products were cleaned using size‐selecting SPRI beads, quantified with a Qubit DNA quantifier, and normalized to be within similar concentration ranges on a per‐plate basis. The cleaned, normalized PCR products were indexed with IDT8 Nextera‐compatible Indexes using a dual combinatorial method. Index PCR reactions contained 2 μL of PCR product, 1 μL each of the i5 and i7 indexes, 5 μL of Qiagen Multiplex PCR Master Mix, and 1 μL molecular grade water for a total volume of 10 μL. The thermal cycling protocol consisted of an initial hot start denaturation step of 95°C for 15 min, followed by 8 cycles of 95°C for 30 s, 55°C for 30 s, and 72°C for 30 s, followed by a final elongation step of 72°C for 5 min. The indexed PCR products were then pooled by PCR plate, cleaned, quantified, normalized, and pooled into a single library that was submitted to the Northwest Genomics Center for sequencing on an Illumina NextSeq platform using a mid 300 cycle kit. The samples were prepared and sequenced in three distinct libraries for developmental and time considerations.

### 2.5 Bioinformatics

Upon receiving the raw sequence files, we decompressed them using the “tar” command in the terminal. Using CLC Workbench for the first two runs, we merged reads from the same samples across the four sequencing lanes. These merged files were exported as forward and reverse reads in fasta format for each sample and replicate. For the third run, we created a custom Bash script to merge our reads from across sequencing lanes. FastQC (Andrews 2010) was employed to assess the quality of the raw reads, and MultiQC (Ewels et al. 2016) was used to summarize the results across all samples. After confirming acceptable quality metrics, we used cutadapt to trim primers and overhangs from both forward and reverse reads for each of the two primer types (trnL and 12S), followed by another round of FastQC and MultiQC to ensure successful trimming. Trimmed reads were then processed through the “DADA2” package in R (Callahan et al. 2016). We plotted the quality profiles of a subset of reads to ensure normal distribution. Reads were filtered and trimmed using DADA2's “filterAndTrim” function, with reads having expected errors greater than EE = 2 being discarded. The “learnErrors” function was used to assess error rates, and the filtered reads were denoised using the “dada” command, which eliminates erroneous reads to reconstruct the true community composition. Forward and reverse reads were merged using “mergePairs”. A sequence table was generated using the “makeSequenceTable” function, and chimeras were removed with the “removeBimeraDenovo” command. The resulting sequence table was converted to a matrix and saved as a CSV file.

### 2.6 NCBI Blast

Given the large number of ornamental and non‐native species in urban areas, creating a comprehensive custom reference database was not practical. The fasta files were uploaded to NCBI's Blastn tool (Madden 2002) for manual review of sequence matches. We identified matches to the lowest taxonomic unit when we had high confidence based on high “Percent Identity” (above 95%) and high “Query Cover” scores (above 90%), and when the species were likely to be found in our study area. For unlikely species, we either classified them at the genus level or assigned a closely related species known to exist in the area. For instance, reads matching European beaver ( *Castor fiber* ) were identified as American beaver ( *Castor canadensis* ). When sequences matched multiple species that shared ≥ 95% similarity, the lowest common taxonomic unit was recorded (largely an issue with shrews and voles). We also documented instances where no sequences matched the NCBI nucleotide database.

### 2.7 Additional Read Filtering

We excluded samples that failed to amplify in at least two PCR replicates or had fewer than 100 total reads. For each sample, we retained only prey items that were detected in at least two out of three PCR replicates and averaged the number of reads for each prey item across those replicates. To account for contamination, we used extraction blanks and PCR negatives, averaging the reads from these controls and subtracting them from the total reads for each sequence in the respective samples. Additionally, we removed any human ( *Homo sapiens* ) reads from the dataset. We then calculated the total number of reads per sample, excluding both contamination and canid reads. Following standard practices, we considered only the reads representing at least 1% of the total for plants and 0.05% for vertebrates, after excluding canid and contamination reads (Caspi, Serrano, et al. 2025). To identify what species were eaten across populations, we included scats that did not successfully genotype, but we excluded scats that had more non‐canid carnivore reads than canid reads because the species of origin typically has the highest read count. Thus, these scats likely originated from a different carnivore species as opposed to being cases where coyotes consumed the other carnivore. In cases where coyotes consumed these carnivores, the scat should have more canid reads than reads of the other carnivore. Consumption of dogs by coyotes could not be identified, however, because the 12S DNA metabarcoding region cannot distinguish among these canids.

### 2.8 Taxonomic Resolution

Plant taxonomic identifications were limited to those that we were confident were from diet items rather than environmental contamination, such as pollen or substrate contamination (Sato 2025, supplemental text). Plant taxa were identified at the genus level when possible and otherwise were identified at the family level. For corn, trnL cannot specify species, but when *Zea* and closely related grass taxa were the closest match in NCBI Blast, we counted these reads as *Zea*. Other *Poaceae* reads that did not have *Zea* as a top match were discarded as contamination unless representing another genus that likely represented a purposefully consumed food item (e.g., *Avena, Hordeum*). Vertebrates were largely identified to species but were represented at the genus level when it was not possible to differentiate between species (e.g., shrews, voles). We ensured that no samples were categorized as containing a species or genus and a higher level classification (genus or family) that could represent the same food item.

### 2.9 Population Dietary Richness

We used frequency of occurrence to understand diet instead of relative read abundance to allow the integration of dietary information across plant and vertebrate primers, because relative read abundance should not be compared across different primers. In addition, frequency of occurrence and relative read abundance values were found to be highly correlated in a study of Pacific fisher ( *Pekania pennanti* ) diets in Washington (Shively et al. 2025). We calculated rarified species richness across all study areas without including individual coyote IDs, resampling to the lowest number of samples per area (*n* = 106 in Bainbridge) and resampling 1000 times.

### 2.10 Individual Dietary Diversity, Richness, and Specialization

**FIGURE 3:**
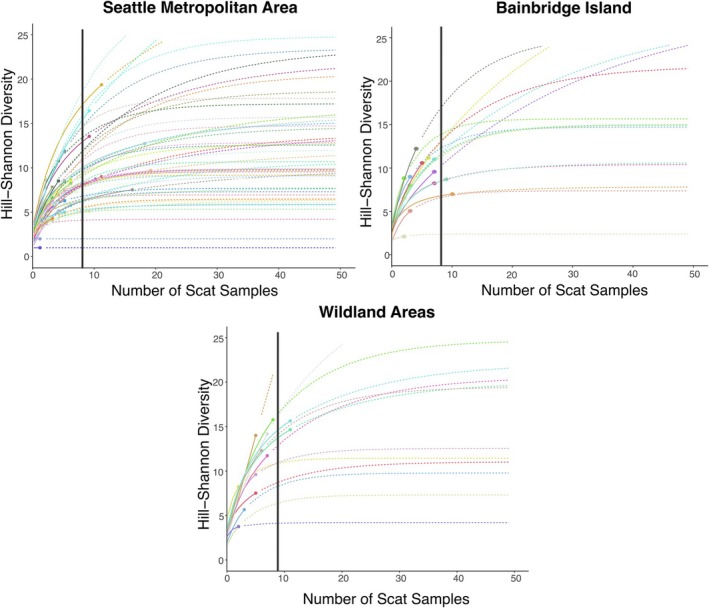
Species accumulation curves for all individuals identified with more than 1 scat sample against Hill‐Shannon Diversity for all three study regions. Each line represents an individual. Where the line is solid is the observed value, the dashed component is interpolated by the “iNEXT” package in R. A vertical black bar is drawn at the 8‐scat cutoff for each graph.

For individual analyses, we limited analyses to individuals with at least 8 scats based on species accumulation curves (Figure 3). While the accumulation curves are not saturated at this point, the trends in the diversity across individuals remain the same. This allows us to increase the number of individuals kept in analyses with accurate diversity trends at the trade‐off of reduced absolute diversity values. We next calculated rarified frequency of occurrence across individuals using the “estimateD” function from the “iNEXT” package in R based on presence/absence of dietary items in each coyotes' scat samples. This function rarefies to the lowest sample size (*n* = 8) to account for differences in diversity related to uneven sampling among individuals (Figure 3). To determine how urbanization affected niche breadth across individuals, we calculated Levin's niche breadth index (*B*) using the rarified frequency of occurrence dataset for each study area and the formula $B=\frac{1}{\Sigma p_{i}^{2}}$ where *p*_*i*_ is the proportion of resources used (Levin 1968). To meet assumptions of this index, we standardized the data so that the frequency of occurrence of diet items summed to one for each individual coyote prior to calculating *B*. We calculated Hill‐species richness and Hill‐Shannon diversity across the diet of individual coyotes using the “estimateD” function from the “iNEXT” package in R based. We used an analysis of variance test (ANOVA) to test for statistical differences in dietary richness and diversity among individuals across study areas.

To assess individual specialization, we computed a Jaccard dissimilarity matrix using the “vegdist” function from the “vegan” package (Oksanen et al. 2022). We then used the “Eindex” function from the “RInSp” package in R (Zaccarelli et al. 2013) to calculate a jackknife cross‐validated estimate of the E index for individual diet specialization among individuals within each population. These indices are derived from the DIETA1 software and based on Araújo et al. (2008). The *E* index measures the degree of individual variation in diet, ranging from 0 (all individuals have identical diets) to 1 (each individual has a unique diet). We used an ANOVA to test for statistical differences in *E* index of specializations and Levin's niche breadth among individuals across study areas. We then used a permutation‐based multivariate analysis of variance (PERMANOVA) on the dissimilarity matrices to determine if diet varied among individuals and among our study areas. PERMANOVA's were conducted in R using the “adonis2” function from the “vegan” package. To account for unequal sampling sizes between individuals, we ran 1000 separate PERMANOVA's drawing eight samples from each individual and averaged across the results.

## 3 Results

### 3.1 Sampling

Of 1220 scats collected in the Seattle metropolitan area, 940 were deemed of coyote origin and successfully sequenced for diet. Of these, 827 were successfully genotyped (*n* = 147 individuals). On average there were 5.59 scats collected per individual (SD = 6.42, range = 1–36), and 35 individuals had at least 8 scat samples ($\bar{x}$ = 14.57, SD = 7.57). Of 145 Bainbridge Island scats collected, 106 were successfully sequenced for diet, and 99 were assigned to one of 26 coyotes (*x* = 3.96 scats per individual, SD = 3.17, range = 1–12 scats). Five individuals had at least 8 scat samples ($\bar{x}$ = 9.4, SD = 1.67). Of 175 scats from our two wildland study areas, 146 were successfully sequenced for diet (*n* = 106 from the Okanagan and 35 from the Northeast). Of these, 105 were successfully genotyped (*n* = 15 individuals), with an average of 9.6 scat samples collected per individual (SD = 4.14, range = 1–18). Ten individuals (7 from OK, 3 from NE) had at least eight samples assigned to them ($\bar{x}$ = 11.8 scat samples/individual, SD = 2.82).

For wildland samples, 54% of samples were collected during the summer. Six out of ten wildland coyotes with 8+ scats from the wildland study areas had the majority of their samples collected during the summer (Table S1). Across all sequenced urban samples, 83% of samples were collected during the summer. Similarly, for individuals with more than 8 scats in the urban study area, 84% of scat samples were collected in the summer. All scat samples were collected during the summer for the rural Bainbridge Island study area. The distribution of samples collected by year across study areas can be found in Table S2.

### 3.2 Population Dietary Richness

**FIGURE 4:**
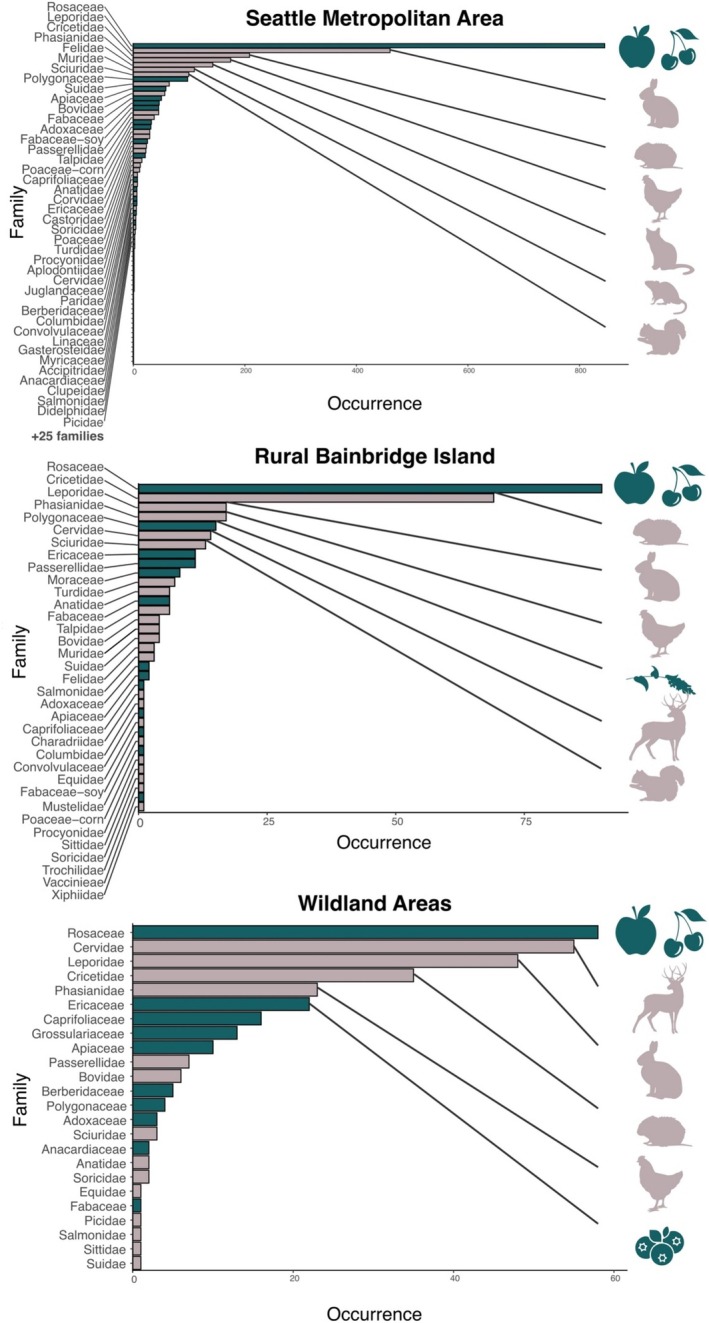
Frequency of occurrence of different taxonomic families identified across all coyote scats for each study region. The top dietary items are depicted with silhouettes to the right of the bars. Vertebrate prey are shown in light brown, while vegetative foods are depicted in dark green. To facilitate visualization, the 25 families with lowest occurrence in the Seattle Metropolitan Area were removed.

**FIGURE 5:**
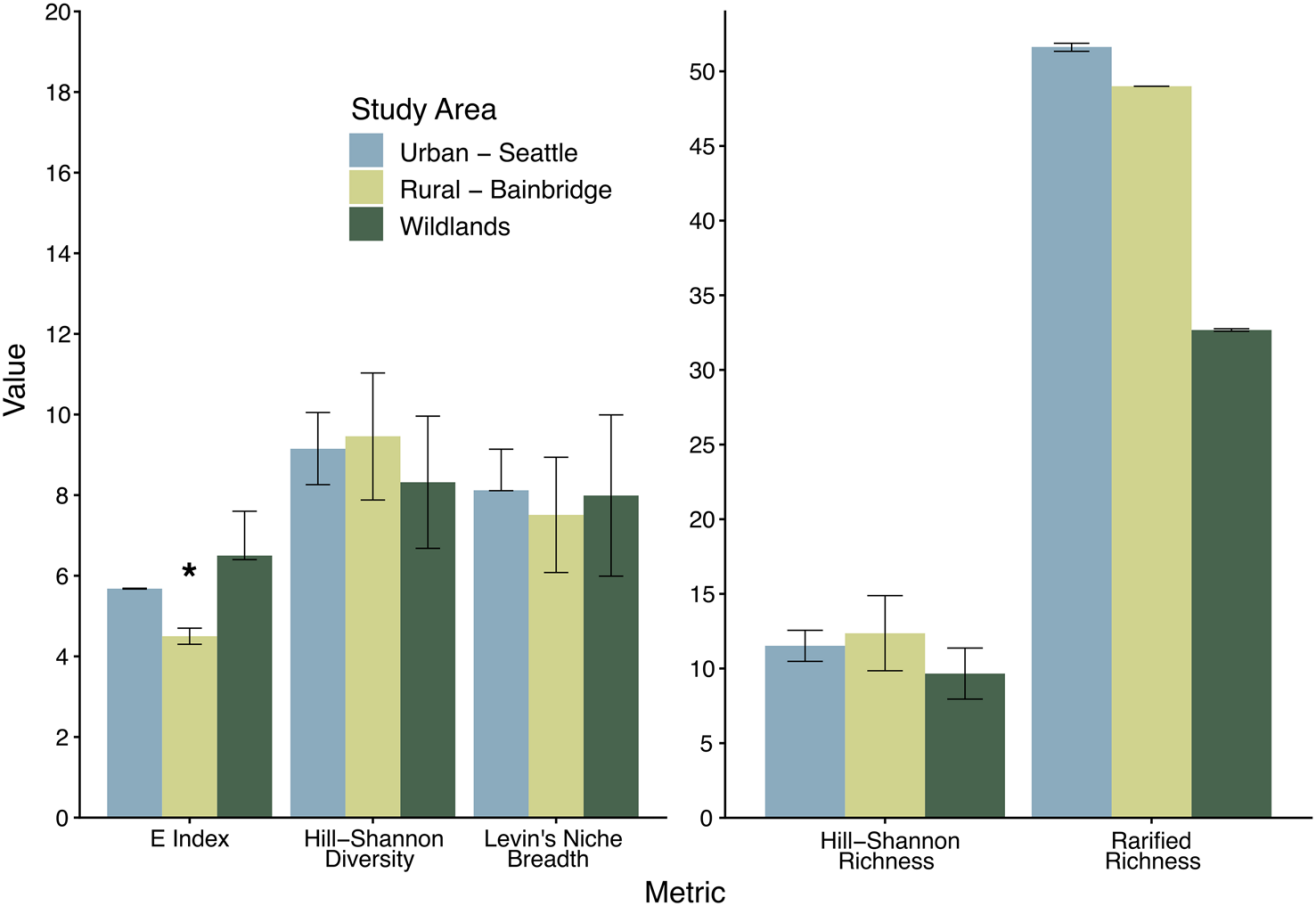
Bar graph of number of average *E*‐index of specialization, Hill‐Shannon diversity, and Levin's niche breadth on the left. *E*‐index ranges from 0 to 1, but is scaled by 10 to facilitate visualization. On the right Hill‐Shannon richness measured across individuals and rarified richness measured across the population. While the other metrics naturally range between 0 and 100. 95% confidence intervals are presented for all metrics. Seattle metropolitan area results are displayed in slate blue, Bainbridge Island in light green, and the wildland areas in dark green. * signifies statistical difference found among study areas.

In the Seattle metropolitan area, we identified dietary items from a total of 65 families (78 genera and species): 42 vertebrate families (40 species; 10 additional genera) and 23 plant families (28 identified genera) were consumed with frequency of occurrence values ≥ 0.01 (Figures 4 and 5, Tables S3 and S4). In the Bainbridge Island study area, we identified items from a total of 33 families: 23 vertebrate families (24 species, 5 additional genera) and 10 plant families (11 identified genera). Lastly, for the wildland study areas we identified items from only 24 families total: 14 vertebrate families (12 species, 4 additional genera) and 10 plant families (10 identified genera) across all of our scat samples. When rarified to control for differences in sample size, population‐level dietary richness was similar between the Seattle metropolitan area ($\bar{x}$ = 51.63, 95% CI: 51.34–51.88) and rural Bainbridge Island (*x* = 49; No CIs as this was un‐rarified), and much lower in the wildland area ($\bar{x}$ = 32.67, 95% CI: 32.59–32.76). Since 46% of wildland scats were collected in winter, we calculated rarified richness between summer and winter in the wildland study system and found slightly elevated richness values in the summer ($\bar{x}$ summer = 27.19, 95% CI: 27.14–27.24; *x* = 23 winter; Table S5).

### 3.3 Individual Dietary Richness, Diversity, and Specialization

PERMANOVAs for each study area revealed significant differences in diet among individuals within each study area (Seattle metropolitan area: $R_{\mathrm{ID}}^{2}$ = 0.33, *p* = 0.001; Bainbridge Island: $R_{\mathrm{ID}}^{2}$ = 0.22, *p* = 0.010; Wildland: $R_{\mathrm{ID}}^{2}$ = 0.29, *p* = 0.001). Based on these PERMANOVAs, 22%–33% of diet variation within each study area was explained by individual coyote ID. Levin's niche breadth index, a measure of individual diet breadth, was similar among all three study areas (Figure 5; Table 1; ANOVA *F*_2,47_ = 0.11, *p* = 0.90), but widest by a slight margin for the Seattle metropolitan area coyotes, followed by the wildland coyotes, and lastly the rural Bainbridge Island coyotes. Despite the differences in population‐level species richness, dietary richness and diversity per individual coyote was remarkably similar among the three study areas (Figure 5; Table 1; diversity *F*_2,47_ = 0.47, *p* = 0.63; richness *F*_2,47_ = 1.83, *p* = 0.17).

**TABLE 1:** Means and 95% confidence intervals (in parenthesis), for different diet‐related metrics.

Study area	Urban	Rural Bainbridge	Wildland	*F*‐statistic (*F*_2_,_*x*_)	Degrees of freedom (numerator, denominator)	*p*
*E*‐Index	0.57 (0.567–0.569)	0.45 (0.43–0.47)	0.65 (0.64–0.67)	888.54	2, 47	< 0.0001
Hill‐Shannon diversity	$\bar{x}$ = 9.15 (8.26–10.05)	9.46 (7.88–11.03)	8.32 (6.68–9.96)	0.47	2, 47	0.63
Hill‐Shannon richness	$\bar{x}$ = 11.52 (10.48–12.56)	12.36 (9.85–14.88)	9.66 (7.95–11.37)	1.83	2, 47	0.17
Levin's niche breadth index	$\bar{x}$ = 8.12 (7.11–9.14)	7.51 (6.08–8.94)	7.99 (5.99–9.99)	0.11	2, 47	0.90

*Note:* ANOVA results comparing metrics across study areas are reported in the last row.

The E index of specialization differed significantly among study regions (Figure 5; Table 1; *F*_2,47_ = 888.54, *p* < 0.0001) and was lowest for Bainbridge Island coyotes, intermediate for Seattle metropolitan area coyotes, and highest for wildland coyotes.

## 4 Discussion

We found surprisingly similar niche widths among coyotes occupying landscapes with varying degrees of urbanization in Washington. Despite similar levels of diet diversity among individuals within a study area, we found evidence for specialization among individuals that varied substantially across these diverse landscape contexts. Specialization was lower in the urban and rural island landscapes than in the wildland areas. While the coyote's success is generally attributed to their adaptability and generalist nature (Hody and Kays 2018), understanding whether their populations are composed of individual generalists or specialists with substantial individual behavioral variation has important management implications across urbanization gradients.

Our wildland study areas had the highest degree of individual specialization. These study areas were much larger (~3000 km^2^ each) than Bainbridge Island (~72 km^2^) and the Seattle metropolitan area (~1200 km^2^), and coyotes likely occurred at much lower densities in the wildland areas (Fischer et al. 2012; Šálek et al. 2014). Coyotes in more urbanized regions have significantly smaller home ranges (Atwood et al. 2010; Šálek et al. 2014; Ward et al. 2018). This constraint may reduce the potential for specialization, especially if abundant prey species like cottontail rabbits are homogenously distributed across the landscape. While we do not know the exact home range size of coyotes in Seattle, coyotes in other urban regions such as Chicago have had recorded home ranges as small as 5 km^2^ (Gehrt et al. 2009), whereas coyotes in our wildland areas had home ranges ~35 km^2^ (*n* = 34 coyotes; Prugh et al. 2023). Likewise, on a relatively small island such as Bainbridge, prey availability is likely to be more homogenous than across a larger wildland area with diverse topography and land cover (Tobler 1970). Thus, home ranges and territoriality may strongly influence coyote diet and access to individual resources throughout our study areas. Our findings suggest that coyotes in more urban areas may face much lower degrees of competition due to prolific anthropogenic resources and abundant non‐native prey despite having elevated interspecific carnivore density and conspecific densities, consistent with other studies of urban carnivores (Rodewald et al. 2011; Fischer et al. 2012). While we do not have estimates of resource availability in our study regions, other works have demonstrated that resource availability is generally higher in urban regions than in non‐urban regions (Hansen et al. 2020; Thompson et al. 2021). The increased specialization among wildland coyotes may reflect increased intraspecific competition due to lower diversity and abundance of resources, as well as increased interspecific competition with other mesocarnivores such as bobcats ( *Lynx rufus* ) and apex predators including wolves ( *Canis lupus* ) and mountain lions (*Puma concolor*, Prugh et al. 2023).

Dietary richness is often greater in urban regions as a result of access to both native and anthropogenic food sources (Murray et al. 2015; de Souza Laurindo and Vizentin‐Bugoni 2020; Larson et al. 2020). However, little research has uncovered if this diversity is reflected at both the population level and at the individual level. While we identified approximately 60% more vertebrate and plant taxa within our urban study system across all individuals, rarified individual diet diversity was only slightly lower in our wildland study areas compared to Bainbridge Island and Seattle metropolitan area coyotes. The lack of difference in individual dietary diversity across regions may further indicate that coyotes are constrained to the resources in their immediate vicinity. With significantly smaller home ranges in urban areas, there may be relatively few prey species available to any given individual as a result of this smaller foraging and hunting area despite the higher diversity of dietary items across the entire urban region. Additionally, seasonality and sample size may influence the overall population‐level taxa richness among the study regions. Most samples for our urban and island study areas were collected during the summer when we would expect plant resources like berries to be more available, whereas 46% our samples for the wildland areas were collected during the winter. However, there were only three plant taxa not identified in the winter that were identified in the summer (Crataegus, Ribes, Trifolium) and one plant that was only identified in the winter (Rhus, Table S5). Rarified richness between seasons in the wildland study system suggest slightly higher diet diversity in the summer (additional ~4 taxa). However, relatively similar values of Hill‐Shannon richness among individuals across all three study systems suggest that these differences should not strongly affect results.

In urban and rural areas where conflict or perceived conflict with coyotes is high (Poessel, Gese, and Young 2017), this dietary individualization has important implications. In areas with higher degrees of population‐wide individual specialization like our wildland areas, removing “problem” individuals who are depredating domestic animals can be more effective than population‐wide culling at reducing conflict (Blejwas et al. 2002). For example, while consumption of anthropogenic foods was rare in our wildland areas, one individual accounted for half of all anthropogenic food consumed (*n* = 4/8 occurrences of anthropogenic items). In other systems, individual predators can have tremendous effects on prey populations. For instance, Fest‐Bianchet et al. (2006) found that individual mountain lions were likely responsible for killing a large portion of a bighorn sheep ( *Ovis canadensis* ) population, leading to major declines. Similarly, extirpation of the Stephen's Island wren ( *Xenicus lyalli* ) was the result of predation by a single domestic cat ( *Felis catus* ; Greenway 1967). In urban areas, actual or perceived coyote consumption of domestic cats and dogs is a major source of human‐coyote conflict, but removal of “problem” coyotes is unlikely to be effective due to low individual specialization among urban coyotes. Although some coyotes in the Seattle area consumed cats more frequently than others (0%–46% of scats per coyote; mean = 15%, SD = 0.15), 74% of coyotes in the Seattle area had cat remains in at least one of their scats (26 of 35 individuals, 74%). Thus, other strategies such as keeping domestic cats indoors or dogs on leashes are essential to promoting co‐existence in cities (Poessel, Mock, and Breck 2017; Poessel, Gese, and Young 2017).

While dietary richness can reveal how varied an individual's diet may be, niche breadth can indicate the dispersion of dietary diversity and point to either widespread consumption of many items or heavy consumption of relatively few taxa. Niche breadth was surprisingly similar among study areas and across individuals. The relatively narrow values for niche breadth compared to the number of resources available to them indicate that coyote diet is largely made up of a few key species, despite finding 64 taxa in the Seattle metropolitan area diet versus 27 taxa across our wildland study areas. For instance, Eastern cottontail rabbits (*Sylvilagus floridianus*) occurred in most scats in the Seattle area, and likewise cervids were widely consumed in the wildland study areas. Across all study areas, coyotes appear to be eating what is easily available to them within their home ranges and acting as dietary generalists at both the individual‐ and population‐level. However, we could not assess actual prey distribution and availability across the landscape. To better understand the relationship between prey availability and niche breadth, further research should be conducted to estimate prey availability.

Because scats represent a snapshot of an individual's consumption, multiple samples from the same individual are required to accurately characterize diets (Prugh et al. 2008). In our case, species accumulation curves indicated that we needed at least 8 scat samples per individual before dietary diversity began to level off. While we likely underestimated absolute dietary richness and diversity, the relative trends remain consistent at this cutoff. Repeated sampling of the same individual can be challenging, especially across large study areas or in areas with high turnover rates. In our wildland study areas, for example, the number of coyotes with at least 8 successfully genotyped scats was limited because fieldwork covered about 3000 km^2^ in each area, while the number of individuals on Bainbridge Island was likely constrained by its small land area. Additionally, while DNA metabarcoding offers a much finer resolution of diet compared to stable isotope analysis and is capable of identifying most vertebrate species and many plant genera, a significant drawback is the need for repeated sampling of individuals. Stable isotope analysis offers inferior taxonomic resolution but has the advantage of integrating dietary information over longer periods from single samples (Crawford et al. 2008; Alberdi et al. 2019; Ando et al. 2020). Future research should consider combining lower‐resolution information such as stable isotope analysis in conjunction with DNA metabarcoding to increase long‐term understanding of dietary trends while being able to accurately understand diet diversity (Bonin et al. 2020).

Understanding dietary specialization and diversity has important ecological and management implications. The patterns of prey consumption by individual predators can alter prey distribution, behavior, and population size (Packer et al. 2003; Salo et al. 2010), which can have cascading effects on the vegetative landscape (Schmitz et al. 2000). Many studies have examined dietary differences between urban and non‐urban populations (Sugden et al. 2021), but few have investigated the individual behaviors and choices that scale up to these population‐level differences or how these differences may arise (Romero‐Vidal et al. 2023; Caspi, Serrano, et al. 2025; Caspi, Sit, et al. 2025). Urban areas can support high densities of mesocarnivores due in large part to abundant resources available to wildlife, yet we have little understanding of how these mesocarnivore densities interplay with abundant resources and shape the landscape of competition. Our results indicate resources in urban areas may be sufficiently abundant that coyote densities are limited by territoriality rather than resources, thereby relaxing the intensity of intraspecific competition and dietary partitioning. This contrasts with Caspi, Sit, et al. (2025) who found increased levels of specialization among individual coyotes in San Francisco, California compared to coyotes in nearby rural landscapes. While lethal removal of coyotes in urban areas can be an effective management tool (Breck et al. 2017), variation in degrees of individual specialization may render this management option ineffective when conflict occurs due to consumption of dietary items that are consumed across the population. This highlights the need for context‐dependent studies on diet for urban species in order to understand when and which management options are likely to mitigate conflicts.

## Author Contributions

**Samantha Erin Sophia Kreling:** conceptualization (equal), data curation (equal), formal analysis (equal), investigation (equal), methodology (equal), validation (equal), visualization (equal), writing – original draft (equal), writing – review and editing (equal). **Ellen M. Reese:** formal analysis (equal), methodology (equal), project administration (equal), supervision (equal), validation (equal), writing – review and editing (equal). **Olivia M. Cavalluzzi:** investigation (equal), methodology (equal), writing – original draft (equal), writing – review and editing (equal). **Alexandra Renouard:** conceptualization (equal), methodology (equal), writing – original draft (equal), writing – review and editing (equal). **Yasmine Hentati:** conceptualization (equal), investigation (equal), methodology (equal), writing – original draft (equal), writing – review and editing (equal). **Robert A. Long:** conceptualization (equal), funding acquisition (equal), methodology (equal), project administration (equal), supervision (equal), writing – original draft (equal), writing – review and editing (equal). **Laura R. Prugh:** conceptualization (equal), formal analysis (equal), funding acquisition (equal), investigation (equal), methodology (equal), project administration (equal), supervision (equal), writing – original draft (equal), writing – review and editing (equal).

## Funding

This study was supported by the National Science Foundation (DEB‐2223973 and DEB‐1652420), Woodland Park Zoo, The Animal Welfare Christine Steven's Wildlife Award, and the University of Washington's Research Royalty Fund, Hall Conservation Genetics Research Award, School of Environmental and Forest Sciences Recruitment Fellowship, and College of Environment's Graduate Research Fellowship.

## Conflicts of Interest

The authors declare no conflicts of interest.

## Supporting information


**Table S1:** Number of scats collected per season (winter = October—March, summer = April–September; based on seasonal changes in our study areas) for each individual coyotes with at least 8 total scats collected.
**Table S2:** Number of scats collected per year across all study areas.
**Table S3:** Taxonomic families present within each study area with frequency of occurrence greater than 0.01.
**Table S4:** Table of higher resolution taxonomy sorted alphabetically within taxa type. Each taxon is listed with its scientific and common name. An “X” denotes presence within that study system while a “—” denotes absence. For the wildland study area deer are listed under *Odocoileus* as both *Odocoileus hemionus* and *Odocoileus virginianus* are present in parts of the wildland study system and are not distinguishable using these methods. On Bainbridge Island and in the Seattle metropolitan area, only *Odocoileus hemionus* occurs.
**Table S5:** Taxa identified in the wildland study areas by season (winter = October—March, summer = April–September; based on seasonal changes in our study areas).

## Data Availability

All the required data are uploaded as Supporting Information for review on Dryad Digital Repository and can be found at this link: https://doi.org/10.5061/dryad.44j0zpcxg.
