# Supplementary material for: Urbanization Drives Patterns of Individual Specialization but Not Dietary Diversity Among Coyotes
Source: Ecol Evol. 2026 Aug 9;16(8):e74170. doi: 10.1002/ece3.74170 (PMC13453434; doi:10.1002/ece3.74170)
Supplement: Supplementary file 1 [file ECE3-16-e74170-s001.docx]

**Supplement**
Table S1: Number of scats collected per season (winter = October – March, summer = April-September; based on seasonal changes in our study areas) for each individual coyotes with at least 8 total scats collected.

| **Individual ID** | **Study Area** | **Summer** | **Winter** |
| --- | --- | --- | --- |
| CoyoteF01 | Urban | 11 | 25 |
| CoyoteF03 | Urban | 27 | 3 |
| CoyoteF05 | Urban | 20 | 0 |
| CoyoteF06 | Urban | 8 | 1 |
| CoyoteF07 | Urban | 9 | 0 |
| CoyoteF08 | Urban | 15 | 0 |
| CoyoteF09 | Urban | 25 | 0 |
| CoyoteF10 | Urban | 12 | 0 |
| CoyoteF22 | Urban | 11 | 0 |
| CoyoteF25 | Urban | 4 | 9 |
| CoyoteF28 | Urban | 8 | 0 |
| CoyoteF35 | Urban | 8 | 20 |
| CoyoteM02 | Urban | 16 | 15 |
| CoyoteM05 | Urban | 24 | 1 |
| CoyoteM07 | Urban | 18 | 0 |
| CoyoteM08 | Urban | 18 | 0 |
| CoyoteM09 | Urban | 7 | 1 |
| CoyoteM10 | Urban | 13 | 0 |
| CoyoteM12 | Urban | 10 | 0 |
| CoyoteM23 | Urban | 12 | 0 |
| CoyoteM24 | Urban | 8 | 4 |
| CoyoteM27 | Urban | 8 | 0 |
| CoyoteM30 | Urban | 8 | 0 |
| CoyoteM33 | Urban | 7 | 1 |
| CoyoteM38 | Urban | 3 | 11 |
| STCoyF06 | Urban | 8 | 0 |
| STCoyF07 | Urban | 6 | 2 |
| STCoyM02 | Urban | 11 | 0 |
| STCoyM03 | Urban | 14 | 0 |
| STCoyM05 | Urban | 10 | 0 |
| STCoyM06 | Urban | 9 | 0 |
| STCoyM07 | Urban | 14 | 0 |
| STCoyM08 | Urban | 7 | 3 |
| STCoyM10 | Urban | 11 | 0 |
| STCoyM12 | Urban | 9 | 0 |
| MVIND36 | Wildland | 1 | 9 |
| MVIND37 | Wildland | 6 | 2 |
| MVIND39 | Wildland | 4 | 14 |
| MVIND51 | Wildland | 0 | 11 |
| MVIND58 | Wildland | 4 | 10 |
| MVIND74 | Wildland | 5 | 5 |
| MVIND79 | Wildland | 10 | 1 |
| NEIND14 | Wildland | 10 | 2 |
| NEIND22 | Wildland | 9 | 0 |
| NEIND24 | Wildland | 13 | 1 |
| BCoyoteF04 | Rural/Island | 9 | 0 |
| BCoyoteF08 | Rural/Island | 8 | 0 |
| BCoyoteM02 | Rural/Island | 8 | 0 |
| BCoyoteM04 | Rural/Island | 10 | 0 |
| BCoyoteM08 | Rural/Island | 12 | 0 |

Table S2: Number of scats collected per year across all study areas.

| Study Area | 2018 | 2019 | 2020 | 2021 | 2022 | 2023 | Year not recorded | Total |
| --- | --- | --- | --- | --- | --- | --- | --- | --- |
| Bainbridge | 0 | 0 | 0 | 106 | 0 | 0 | 0 | 106 |
| Seattle Area | 0 | 0 | 23 | 477 | 317 | 122 | 1 | 940 |
| Wildlands | 3 | 24 | 74 | 28 | 0 | 0 | 17 | 146 |

Table S3: Taxonomic families present within each study area with frequency of occurrence greater than 0.01.

| **Scientific Name** | **Taxa Type** | **Common Name** | **Seattle Metropolitan Area FOO** | **Bainbridge Island FOO** | **Okanagan FOO** | **Northeast FOO** | **Wildland Combined FOO** |
| --- | --- | --- | --- | --- | --- | --- | --- |
| Accipitridae | Bird | Hawks | 0.01 | -- | -- | -- | -- |
| Adoxaceae | Plant | Elderberry Family | 0.05 | 0.02 | 0.02 | -- | 0.02 |
| Anacardiaceae | Plant | Cashew Family | 0.01 | -- | 0.02 | -- | 0.01 |
| Anatidae | Bird | Ducks | 0.03 | 0.06 | 0.02 | -- | 0.01 |
| Apiaceae | Plant | Carrot Family | 0.06 | 0.02 | 0.09 | -- | 0.07 |
| Aplodontidae | Mammal | Mountain Beavers | 0.01 | -- | -- | -- | -- |
| Berberidaceae | Plant | Barberry Family | 0.01 | -- | 0.02 | 0.09 | 0.04 |
| Bovidae | Mammal | Cattle | 0.06 | 0.04 | 0.06 | -- | 0.04 |
| Caprifoliaceae | Plant | Honeysuckle Family | 0.03 | 0.01 | 0.08 | 0.23 | 0.11 |
| Castoridae | Mammal | Beavers | 0.03 | -- | -- | -- | -- |
| Cervidae | Mammal | Deer & Elk | 0.01 | 0.13 | 0.28 | 0.71 | 0.38 |
| Charadriidae | Bird | Plovers & Lapwings | -- | 0.01 | -- | -- | -- |
| Clupeidae | Fish | Herrings & Sprats | 0.01 | -- | -- | -- | -- |
| Columbidae | Bird | Doves & Pigeons | 0.01 | 0.01 | -- | -- | -- |
| Convolvulaceae | Plant | Morning Glory Family | 0.01 | 0.01 | -- | -- | -- |
| Corvidae | Bird | Crows | 0.03 | -- | -- | -- | -- |
| Cricetidae | Mammal | Voles | 0.22 | 0.65 | 0.30 | 0.09 | 0.24 |
| Equidae | Mammal | Horses | -- | 0.01 | 0.01 | -- | 0.01 |
| Ericaceae | Plant | Heath Family | 0.03 | 0.10 | 0.15 | 0.14 | 0.15 |
| Fabaceae | Plant | Legume Family | 0.05 | 0.06 | -- | 0.03 | 0.01 |
| Fabaceae – Soy | Plant | Soy | 0.05 | 0.01 | -- | -- | -- |
| Felidae | Mammal | Cats | 0.15 | 0.03 | -- | -- | -- |
| Gasterosteidae | Fish | Stickleback | 0.01 | -- | -- | -- | -- |
| Grossulariaceae | Plant | Currant Family | -- | -- | 0.05 | 0.23 | 0.09 |
| Juglandaceae | Plant | Walnut Family | 0.01 | -- | -- | -- | -- |
| Leporidae | Mammal | Rabbits | 0.49 | 0.16 | 0.32 | 0.37 | 0.33 |
| Linaceae | Plant | Flax Family | 0.01 | -- | -- | -- | -- |
| Moraceae | Plant | Mulberry Family | -- | 0.08 | -- | -- | -- |
| Muridae | Mammal | Rats | 0.12 | 0.04 | -- | -- | -- |
| Mustelidae | Mammal | Weasels | -- | 0.01 | -- | -- | -- |
| Myricaceae | Plant | Sweet Gale Family | 0.01 | -- | -- | -- | -- |
| Paridae | Bird | Tits & Chickadees | 0.01 | -- | -- | -- | -- |
| Passerellidae | Bird | Passerines | 0.05 | 0.10 | 0.06 | 0.03 | 0.05 |
| Phasianidae | Bird | Pheasants, Chickens, & Turkeys | 0.18 | 0.16 | 0.09 | 0.37 | 0.16 |
| Picidae | Bird | Woodpeckers | -- | -- | 0.01 | -- | 0.01 |
| Poaceae | Plant | Grass Family | 0.02 | -- | -- | -- | -- |
| Poaceae – Corn | Plant | Corn | 0.03 | 0.01 | -- | -- | -- |
| Polygonaceae | Plant | Buckwheat Family | 0.10 | 0.14 | 0.03 | 0.03 | 0.03 |
| Procyonidae | Mammal | Raccoons | 0.01 | 0.01 | -- | -- | -- |
| Rosaceae | Plant | Rose Family | 0.89 | 0.85 | 0.26 | 0.83 | 0.40 |
| Salmonidae | Fish | Salmon | -- | 0.03 | 0.01 | -- | 0.01 |
| Sciuridae | Mammal | Squirrels | 0.11 | 0.12 | 0.03 | -- | 0.02 |
| Sittidae | Bird | Nuthatches | -- | 0.01 | 0.01 | -- | 0.01 |
| Soricidae | Mammal | Shrews | 0.02 | 0.01 | 0.02 | -- | 0.01 |
| Suidae | Mammal | Pigs | 0.07 | 0.04 | 0.01 | -- | 0.01 |
| Talpidae | Mammal | Moles | 0.04 | 0.06 | -- | -- | -- |
| Trochilidae | Bird | Hummingbirds | -- | 0.01 | -- | -- | -- |
| Turdidae | Bird | Robins & Thrushes | 0.02 | 0.07 | -- | -- | -- |
| Xiphiidae | Fish | Swordfishes | -- | 0.01 | -- | -- | -- |

Table S4: Table of higher resolution taxonomy sorted alphabetically within taxa type. Each taxon is listed with its scientific and common name. An ‘X’ denotes presence within that study system while a ‘—’ denotes absence. For the wildland study area deer are listed under *Odocoileus* as both *Odocoileus hemionus* and *Odocoileus virginianus* are present in parts of the wildland study system and are not distinguishable using these methods. On Bainbridge Island and in the Seattle metropolitan area, only *Odocoileus hemionus* occurs.

| **Taxa Type** | **Scientific Name** | **Common Name** | **Seattle Metropolitan Area** | **Bainbridge Island** | **Wildland Combined** |
| --- | --- | --- | --- | --- | --- |
| **Bird** | *Aix sponsa* | Wood Duck | **X** | **—** | **—** |
|  | *Anas* | Ducks | **X** | **X** | **X** |
|  | *Bonasa umbellus* | Ruffed Grouse | **—** | **—** | **X** |
|  | *Catharus ustaulatus* | Swainson’s Thrush | **X** | **—** | **—** |
|  | *Colaptes auratus* | Northern Flicker | **X** | **—** | **X** |
|  | *Columbidae* | Doves | **X** | **—** | **—** |
|  | *Corvus* | Crows | **X** | **—** | **—** |
|  | *Coturnix japonica* | Japanese Quail | **X** | **—** | **—** |
|  | *Dendragapus fuliginosus* | Sooty Grouse | **—** | **—** | **X** |
|  | *Gallus gallus* | Domestic Chicken | **X** | **X** | **—** |
|  | *Ixoreus naevius* | Varied Thrush | **—** | **X** | **—** |
|  | *Junco hyemalis* | Dark-eyed Junco | **X** | **X** | **X** |
|  | *Meleagris gallopavo* | Turkey | **X** | **—** | **X** |
|  | *Neurotrichus gibbsii* | American Shrew Mole | **X** | **X** | **—** |
|  | *Paridae* | Tits | **X** | **—** | **—** |
|  | *Phasianus colchicus* | Ring-necked Pheasant | **X** | **—** | **—** |
|  | *Pipilo maculatus* | Spotted Towhee | **X** | **X** | **X** |
|  | *Poecile Atricapilla* | Black-capped Chickadee | **X** | **—** | **—** |
|  | *Regulus* | Kinglets | **X** | **—** | **—** |
|  | *Sittidae* | Nuthatches |  | **X** | **X** |
|  | *Sturnus vulgaris* | Common Starling | **X** | **—** | **—** |
|  | *Trochilidae* | Hummingbirds | **X** | **—** | **—** |
|  | *Troglodytes* | Wrens | **X** | **—** | **—** |
|  | *Turdus* | True Thrushes | **X** | **X** | **—** |
| **Fish** | *Ameiurus* | Bullheads | **X** | **—** | **—** |
|  | *Brevoortia* | Brevoortia | **X** | **—** | **—** |
|  | *Cyprinus* | Carps | **X** | **—** | **—** |
|  | *Gasterosteus aculeatus* | Three-spined Stickleback | **X** | **—** | **—** |
|  | *Micropterus salmoides* | Large-mouth Bass | **X** | **—** | **—** |
|  | *Oncorhynchus* | Pacific Salmon & Trout | **—** | **X** | **—** |
|  | *Oncorhynchus kisutch* | Coho Salmon | **—** | **—** | **X** |
|  | *Perca* | Perch | **X** | **—** | **—** |
|  | *Xiphias gladius* | Swordfish | **—** | **X** | **—** |
| **Mammal** | *Alces alces* | Moose | **—** | **—** | **X** |
|  | *Aplodontia rufa* | Mountain Beaver | **X** | **—** | **—** |
|  | *Bos taurus* | Domestic Cattle | **X** | **—** | **X** |
|  | *Castor canadensis* | North American Beaver | **X** | **—** | **—** |
|  | *Clethrionomys* | Slender Voles | **—** | **—** | **X** |
|  | *Cricetidae* | Voles | **X** | **X** | **X** |
|  | *Equus caballus* | Domestic Horse | **—** | **—** | **X** |
|  | *Felis catus* | Domestic Cat | **X** | **X** | **—** |
|  | *Lepus americanus* | Snowshoe Hare | **—** | **—** | **X** |
|  | *Microtus longicaudus* | Long-tailed vole | **X** | **—** | **X** |
|  | *Mus musculus* | House Mouse | **X** | **—** | **—** |
|  | *Neogale vison* | American Mink | **—** | **X** | **—** |
|  | *Odocoileus* | Deer (Both black and white tail occur in parts of wildland study areas) | Odocoileus hemionus | Odocoileus hemionus | **X** |
|  | *Odocoileus hemionus* | Black-tailed Deer | **X** | **X** | **—** |
|  | *Oryctolagus cuniculus* | European Rabbit | **X** | **—** | **—** |
|  | *Ovis aries* | Domestic Sheep | **X** | **—** | **—** |
|  | *Procyon lotor* | Raccoon | **X** | **—** | **—** |
|  | *Rattus norvegicus* | Norway Rat | **X** | **X** | **—** |
|  | *Rattus rattus* | Black Rat | **X** | **X** | **—** |
|  | *Scanapus orarius* | Coast Mole | **X** | **—** | **—** |
|  | *Scanapus townsendii* | Townsend’s Mole | **X** | **—** | **—** |
|  | *Sciurus carolinensis* | Eastern Gray Squirrel | **X** | **X** | **—** |
|  | *Sorex* | Long-tailed Shrews | **X** | **X** | **—** |
|  | *Sus scrofa* | Domestic Pig | **X** | **X** | **—** |
|  | *Sylvilagus floridianus* | Eastern Cottontail | **X** | **X** | **—** |
|  | *Tamiasciurus douglasii* | Douglas Squirrel | **X** | **—** | **X** |
| **Plant** | *Actinidia* | Kiwi | **X** | **—** | **—** |
|  | *Anacardium* | Cashews | **X** | **—** | **—** |
|  | Apiaceae | Carrot Family | **X** | **X** | **—** |
|  | *Arachis* | Peanuts | **X** | **—** | **—** |
|  | *Avena* | Oats | **X** | **—** | **—** |
|  | Berberidaceae | Barberry Family | **X** | **—** | **X** |
|  | *Brassica* | Brassicas | **X** | **—** | **—** |
|  | *Cornus* | Dogwoods | **X** | **—** | **—** |
|  | *Crataegus* | Hawthorns | **X** | **—** | **X** |
|  | Ericaceae | Heath Family | **X** | **X** | **—** |
|  | Fabaceae | Legume Family | **X** | **—** | **—** |
|  | Fabaceae – Soy | Soy, Glycine | **X** | **—** | **—** |
|  | *Ipomoea* | Sweet Potatoes | **X** | **X** | **—** |
|  | Juglandaceae | Walnut Family | **X** | **—** | **—** |
|  | *Lactuca* | Lettuces | **X** | **—** | **—** |
|  | *Lens* | Lentils | **X** | **—** | **—** |
|  | *Linum* | Flaxes | **X** | **—** | **—** |
|  | *Morus* | Mulberries | **X** | **X** | **—** |
|  | *Musa* | Bananas | **X** | **X** | **—** |
|  | *Oryza* | Rice | **X** | **—** | **—** |
|  | *Piper* | Pepper | **X** | **—** | **—** |
|  | Poaceae – Corn | Corn | **X** | **—** | **—** |
|  | *Prunus* | Plums | **X** | **X** | **X** |
|  | *Pyrus* | Pears | **X** | **—** | **—** |
|  | *Rhus* | Sumacs | **—** | **—** | **X** |
|  | *Ribes* | Currants | **X** | **—** | **X** |
|  | *Rubus* | Brambles | **X** | **X** | **X** |
|  | *Rumex* | Dock | **X** | **X** | **X** |
|  | Rutaceae | Citrus Family | **X** |  | **—** |
|  | *Sambucus* | Elderberries | **X** | **X** | **X** |
|  | *Sorghum* | Sorghums | **X** | **—** | **—** |
|  | *Symphoricarpos* | Snowberries | **X** | **—** | **X** |
|  | *Theobroma* | Chocolate | **X** | **—** | **—** |
|  | *Trifolium* | Clovers | **X** | **X** | **X** |
|  | *Vaccinium* | Huckleberries | **—** | **—** | **X** |
|  | *Viburnum* | Honeysuckles | **X** | **—** | **—** |
| **Reptile** | *Trachemys scripta* | Red-eared Slider | **X** | **—** | **—** |

Table S5: Taxa identified in the wildland study areas by season (winter = October – March, summer = April-September; based on seasonal changes in our study areas).

| Taxa | Summer | Winter |
| --- | --- | --- |
| **Plant** |  |  |
| Apiaceae | X | X |
| Berberidaceae | X | X |
| Crataegus | X |  |
| Prunus | X | X |
| Rhus |  | X |
| Ribes | X |  |
| Rubus | X | X |
| Rumex | X | X |
| Sambucus | X | X |
| Symphoricarpos | X | X |
| Trifolium | X |  |
| Vaccinium | X | X |
| **Vertebrate** |  |  |
| Alces alces |  | X |
| Anas |  | X |
| Anatidae |  | X |
| Bonasa umbellus | X | X |
| Bos taurus | X | X |
| Clethrionomys | X | X |
| Colaptes auratus | X |  |
| Cricetidae | X | X |
| Dendragapus fuliginosus | X |  |
| Equus caballus |  | X |
| Junco hyemalis | X |  |
| Leporidae | X | X |
| Lepus americanus | X | X |
| Meleagris gallopavo | X |  |
| Microtus longicaudus | X | X |
| Odocoileus | X | X |
| Oncorhynchus kisutch |  | X |
| Passerellidae | X |  |
| Pipilo maculatus | X |  |
| Sittidae |  | X |
| Sorex | X |  |
| Sus scrofa | X |  |
| Tamiasciurus | X |  |

Supplemental Text – Plant taxonomic justification:

Scat samples were collected from the environment where they were subject to both ground and air-based contamination by plants. We used a combination of knowledge of our study area, plant life history traits, and previous coyote diet studies to assign plants as either ‘food’ or ‘not food’. For instance, plant genera that were typically not found to be eaten by coyotes in other studies, had difficult to obtain edible components, and were wind pollinated such as pines were considered not food. We also hypothesized that some wind pollinated plants such as *Rumex* (docks) or *Rhus* (sumacs) were likely food plants given the high availability on the landscape and high nutritional value of edible components. Plants that had high availability of fruits and seeds in our study area were also considered food items (e.g., *Rubus, Vaccinium, Symphoricarpos*). Likewise, plants that represented dietary items likely sourced from anthropogenic food were included even if plants in those genera are not common on the landscape such as *Theobroma* (cacao), *Arachis* (peanuts, a common bird food in our region), or *Musa* (bananas). Finally, some genera of plants were considered nonfood items when they were common on the landscape, but produce limited edible components in our landscape. For example, beaked hazelnut (*Corylus cornuta*) is a common wind-pollinated tree in the Seattle area, but nuts are typically out of reach of coyotes and quickly picked over by other wildlife species such as eastern gray squirrels and are thus unlikely to fall to the ground (King Conservation District Staff). It is likely that some species that were considered non-food were actually eaten by coyotes and that some species considered food may have been contamination. However, we have screened plant taxa as accurately as possible to identify true dietary items for coyotes across our study areas.
